# Some Soybean Cultivars Have Ability to Induce Germination of Sunflower Broomrape

**DOI:** 10.1371/journal.pone.0059715

**Published:** 2013-03-27

**Authors:** Wei Zhang, Yongqing Ma, Zhong Wang, Xiaoxin Ye, Junfeng Shui

**Affiliations:** 1 College of Resources and Environment, Northwest A&F University, Yangling, Shaanxi Province, China; 2 The State Key Laboratory of Soil Erosion and Dryland Farming in the Loess Plateau, Institute of Soil and Water Conservation, Northwest A&F University, Yangling, Shaanxi Province, China; 3 College of Forestry, Northwest A&F University, Yangling, Shaanxi Province, China; University College London, United Kingdom

## Abstract

Sunflower broomrape is a noxious parasitic weed which has caused severe damage to crop ecosystems. Trap crops can release a mixture of allelochemicals to induce the germination of sunflower broomrape. We studied the allelopathic effects of soybean on sunflower broomrape. Fourteen common soybean cultivars were grown in pots. Samples were collected from soybean plants and rhizosphere soil at five growth stages (V1, V3, V5, R2, and R4). The allelopathic effects of soybean reached highest at the V3 stage. Methanolic extracts of soybean roots induced higher broomrape germination than methanolic extracts of stems or leaves. The germination rates induced by root extracts (10-fold dilution) were positively correlated with germination rates induced by stem (10-fold dilution) and leaf extracts (10-fold dilution). The broomrape germination rates induced by root extracts were also positively correlated with soybean nodule diameter and dry weight. The results indicated that soybeans could induce sunflower broomrape germination. We conclude that soybean has the potential to be used as a trap crop for sunflower broomrape.

## Introduction

Parasitic plants take up water and/or nutrients from their host plants, often resulting in a reduction in host plant growth. Parasitic weed infestations cause significant crop loss in many countries [1]–[4]. Broomrapes (*Orobanche* spp.) are root holoparasites lacking chlorophyll. Several broomrape species are weedy, causing severe yield losses in important agricultural crops such as sunflower (*Helianthus annuus* L.), tomato (*Lycopersicon esculentum* Miller), lentils (*Lens culinaris* Medic.), broad beans (*Vicia faba* L.), canola (*Brassica campestris* L.), and melon (*Cucumis melo* L.) [5], [6].

Researchers have proposed a number of methods for controlling broomrape. These methods include hand-weeding, adjustment of sowing dates, herbicide application, selection and breeding of resistant crop cultivars, and use of trap or catch crops [7]–[12]. Among these methods, the use of trap crops is most promising. Trap crops induce broomrape germination without being parasitized themselves. The broomrape seedlings die for lack of nutritional support, a process termed “suicide germination”, thus reducing the broomrape seed bank.

Broomrape seeds require chemical stimulants from hosts (catch crops) or non-hosts (trap crops) to germinate [1]. Strigolactones are a group of compounds that trigger germination of *Striga* spp. Most strigolactones are also able to induce broomrape germination. Strigolactones were first isolated from root exudates of non-host plant cotton [13]. Since then, the ability to produce strigolactones has been observed in many other plant species, including both hosts and non-hosts of *Striga*. Orobanchyl acetate which is one of the strigolactones, was identified from soybean root exudates [14]–[16]. Intercropping soybean with maize (*Zea mays* L.) can reduce the parasitism rate of *Striga hermonthica* (Del.) Benth, resulting in increased maize yield [17].

Many studies have tested the allelopathic effects of soybean. For example, soybean leaf extracts inhibited speargrass (*Imperata cylindrical* L.) germination and growth [18]. Undiluted soybean root exudates promoted cucumber (*Cucumis sativus* L.) germination, whereas diluted exudates inhibited cucumber germination [19]. In regard to their effect on microorganisms, soybean root exudates promoted the growth of *Fusarium semitectum*, *Gliocladium roseum,* and *Fusarium oxysporum* [20]. Little is known about the allelopathic effects of different soybean cultivars on sunflower broomrape. We conducted pot and field studies whose objectives were to (1) compare the allelopathic effects of fourteen soybean cultivars on sunflower broomrape, (2) assay the allelopathic effects of rhizosphere soil, rhizosphere soil extracts, root extracts, stem extracts, and leaf extracts, and (3) determine if the allelopathic effects of soybean changes during the growing season. We also examined the relationship between the physiological characteristics of soybean nodules and allelopathic effects.

## Materials and Methods

### Experiment 1: Pot Experiment

#### Plant and soil materials

Seeds of 14 soybean cultivars (Kenjiandou 36, Suinong 99, Beidou 18, Suinong 10, Fengdou 3, Heinong 28, Dongdou 339, Zhonghuang 13, Hefeng 55, Kenjiandou 35, Heinong 44, Nongda 555, Kenfeng 16, and Ribenchun 95) were purchased from a seed company in Shuangyashan City, Heilongjiang Province, China. Sunflower broomrape seeds were collected in 2010, from under sunflower fields and the field is not privately owned field, in the Xinjiang Uygur Autonomous Region, China.

Soil for the pot experiment was collected from the surface horizon (0–20 cm) of a cultivated field belonging to Guyuan Ecological Station of the Institute of Soil and Water Conservation (35° 99′ N, 106° 44′ E), near Hechuan Village, Guyuan City, Ningxia Hui Autonomous Region, China, and no specific permits were required for this field study. The soil, which is classified as a common dark loessial soil, has the following characteristics: pH, 7.41; soil organic matter, 9.41 g kg^−1^; total N, 0.28 g kg^−1^; available P, 2.28 mg kg^−1^; and available K, 206 mg kg^−1^. The soil was thoroughly mixed and then 8 kg of dry soil was placed in 7.8 L plastic pots. Ten seeds of each cultivar were sown per pot. There were 15 pots per cultivar. The soybean population was thinned to five seedlings per pot after emergence. The pots were placed outside in a sunny area and the plants watered daily. Three pots of each cultivar were destructively sampled at the V1 (Completely unrolled leaf at the unifoliolate node), V3 (Three nodes on the main stem beginning with the unifoliolate node), V5 (Five nodes on the main stem beginning with the unifoliolate node), R2 (Flower at node immediately below the uppermost node with a completely unrolled leaf) and R4 (Pod 2 cm long at one of the four uppermost nodes with a completely unrolled leaf) stages [21]. On each sample date, the pots were cut open and samples of rhizosphere soil were removed. Samples of the leaves, stems, and roots were collected and prepared for extraction as described below [22].

#### Extraction method and germination assay

Sunflower broomrape seeds were surface sterilized by immersion in 1% sodium hypochlorite (v/v) for 3 min and then in 75% methanol (v/v) for 3 min. The seeds were thoroughly rinsed with autoclaved distilled water, and then dried on a clean bench. Glass fiber filter paper disks (8 mm diam., Whatman GF/A) were uniformly laid on moist filter paper (9 cm diam., Shuangquan GB/T1914–2007, Hangzhou Wohua Filter Paper Co., Ltd, China), and then 40–50 sunflower broomrape seeds were put on each glass fiber disk. The disks were dabbed with filter paper to remove excess water prior to application of the extracts.

We assayed the ability of both rhizosphere soil and rhizosphere soil extracts to induce sunflower broomrape germination. For the first assay, 5 g of rhizosphere soil plus 1.5 mL distilled water were spread evenly across the bottoms of Petri dishes (3.5 cm diam.) [23]. Five glass fiber disks with 40–50 sunflower broomrape seeds were put on the soil surface and then the Petri dishes were sealed and incubated at 25°C for 10 days. For the second assay, 5 g of rhizosphere soil plus 10 mL distilled water or methanol were ultrasonic treated for 30 min at 25°C, 50,000 Hz and 300 W in a ultrasonic cleaner (CS-500EII, Ningbo Jiangnan Instrument Factory, Ningbo, China) and then filtered. The filtrates are hereafter referred to as the undiluted rhizosphere soil extracts (0.5 g mL^−1^). The undiluted extracts were diluted 10- or 100-fold with either autoclaved distilled water or methanol for use in the germination assays described below. The diluted solutions are referred to as 10-fold dilution (0.05 g mL^−1^) and 100-fold dilution (0.005 g mL^−1^).

Soybean plant tissues were freeze-dried, and then milled to pass through a 0.35 mm sieve. Plant tissue samples (1.0 g) and 1 mL methanol were added to 1.5 mL centrifuge tubes. The samples were ultrasonic treated for 30 min and then centrifuged at 6400 rpm for 2 min by a centrifugal machine (Millipore Cat. No. XX42 CF0, 60 Lot No. N8JMB042A, Nihon Millipore Ltd. Yonezawa, Japan). The supernatants are hereafter referred to as the undiluted extracts (1 g mL^−1^). These solutions were diluted 10- and 100-fold with either autoclaved distilled water or methanol. The diluted solutions are referred to as 10-fold dilution (0.1 g mL^−1^) and 100-fold dilution (0.01 g mL^−1^).

For the methanolic extracts, allelopathic effects were assayed by applying methanolic extracts (40 µL) to one glass fiber disk in Petri dishes. The dishes were left open for 30 min at room temperature to allow the methanol to evaporate. A second glass fiber disk with sunflower broomrape seeds was placed on the top of the first glass fiber disk. A 40 µL of autoclaved distilled water was added to the former two glass fiber disks. And for the distilled water extracts, allelopathic effects were assayed by applying aqueous extracts (20 µL) to each glass fiber disk with sunflower broomrape seeds in Petri dishes only. Individual treatments were replicated four times. The Petri dishes were then sealed and incubated at 25°C. Germinated and non germinated sunflower broomrape seeds were counted under a binocular dissecting microscope at 20× magnifacation after ten days to assess the germination rate. Sunflower broomrape seeds treated with GR24 (a synthetic analog of strigolactones, provided by Binne Zwanenburg, Radboud University Nijmegen, the Netherlands.) and distilled water were used as positive and negative controls.

#### Soybean nodule diameter and dry weight measurement

In the pot experiment, diameter per nodule of soybean and average nodule dry weight per plant were measured at V1, V3, V5, R2 and R4 stage. For each pot, three plants in each pot were randomly selected for nodule diameter and dry weight measurement. Diameters of all the nodules of the selected plants were measured by vernier caliper and the diameter per nodule of one plant was calculated. Then, all the nodules of one individual plant were put together and dried at 105°C for 0.5 h before dried to constant weight at 80°C. Dry weight of the nodules of each plant was weighted by balance.

### Experiment 2: Field Experiment

Three soybean cultivars (Fengdou 3, Zhonghuang 13 and Kenfeng 16) were planted in field plots at the Institute of Soil and Water Conservation (34° 27′ N, 108° 07′ E), Chinese Academy of Science, Yangling, China and no specific permits were required for this field study. The experimental design was a randomized complete block with three replications. The soil, which is classified as a Lou soil (Typ-Eum-Orthic Anthrosol), has the following properties: pH, 7.98; soil organic matter, 14.0 g kg^−1^; available N, 71.3 mg kg^−1^; available P, 24.2 mg kg^−1^; and available K, 166 mg kg^−1^. Samples were collected from the soybean plants and rhizosphere soil at V3, V5, and R4 stages. The extraction methods and germination assays were the same as described for Experiment 1.

### Data Analysis

Analysis of variance was performed using SPSS 13.0 software (SPSS Inc., Chicago, U.S.). Duncan’s multiple range test was used to separate the means. Correlation analysis was performed to determine whether there were relationships in allelopathic effects among different parts of the soybean plants. Correlations were also performed between the allelopathic effects of roots and the diameter or dry weight of soybean root nodules.

## Results

The germination rate of sunflower broomrape seeds treated with GR24 ranged between 70 and 80%. Distilled water did not induce sunflower broomrape germination.

### Experiment 1

#### Soybean rhizosphere soil and rhizosphere soil extracts

**Figure 1:**
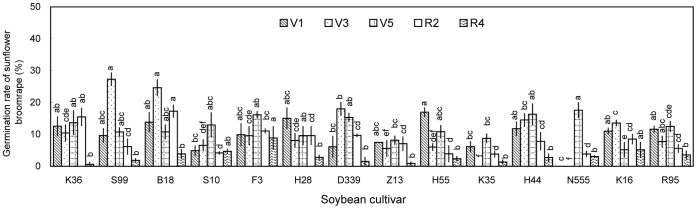
Induction of sunflower broomrape germination by soybean rhizosphere soil in pot experiment. The rhizosphere soil was collected from pot-grown plants at the (A) V1, (B) V3, (C) V5, (D) R2, and (E) R4 stages. Error bars represent the standard error of the mean. Different small letters above the error bars indicate significant differences at the 0.05 level (ANOVA and Duncan’s multiple range test). If any letter marked in one treatment is the same as the other one (in same color column), it indicates no significant differences between them. Abbreviations: K36, Kenjiandou 36; S99, Suinong 99; B18, Beidou 18; S10, Suinong 10; F3, Fengdou 3; H28, Heinong 28; D339, Dongdou 339; Z13, Zhonghuang 13; H55, Hefeng 55; K35, Kenjiandou 35; H44, Heinong 44; N555, Nongda 555; K16, Kenfeng 16; R95, Ribenchun 95.

Sunflower broomrape germination was induced by contact with rhizosphere soil; however, the germination rates were all less than 30% (Fig. 1). The differences among the cultivars were significant (*P*<0.05), with rhizosphere soil from Suinong 99 (27.3%) and Beidou 18 (24.6%) inducing the highest germination rates.

**Figure 2:**
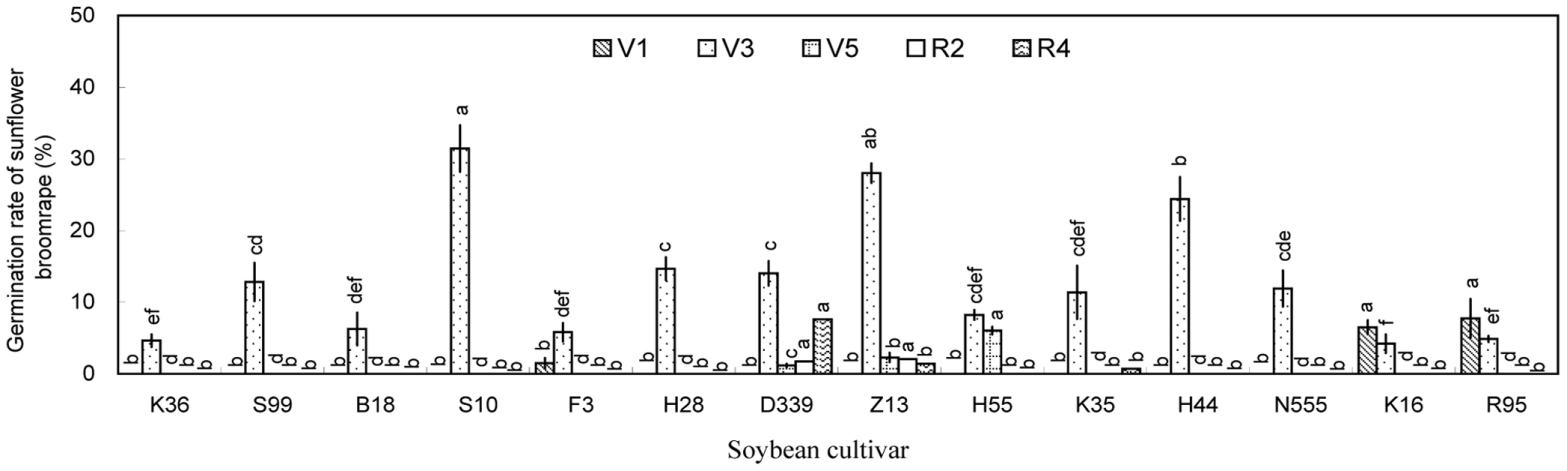
Induction of sunflower broomrape germination by distilled water extracts of soybean rhizosphere soil in pot experiment. The rhizosphere soil was collected from pot-grown plants at the (A) V1, (B) V3, (C) V5, (D) R2, and (E) R4 stages. Error bars represent the standard error of the mean. Different small letters above the error bars indicate significant differences at the 0.05 level (ANOVA and Duncan’s multiple range test). If any letter marked in one treatment is the same as the other one (in same color column), it indicates no significant differences between them. Abbreviations: K36, Kenjiandou 36; S99, Suinong 99; B18, Beidou 18; S10, Suinong 10; F3, Fengdou 3; H28, Heinong 28; D339, Dongdou 339; Z13, Zhonghuang 13; H55, Hefeng 55; K35, Kenjiandou 35; H44, Heinong 44; N555, Nongda 555; K16, Kenfeng 16; R95, Ribenchun 95.

**Figure 3:**
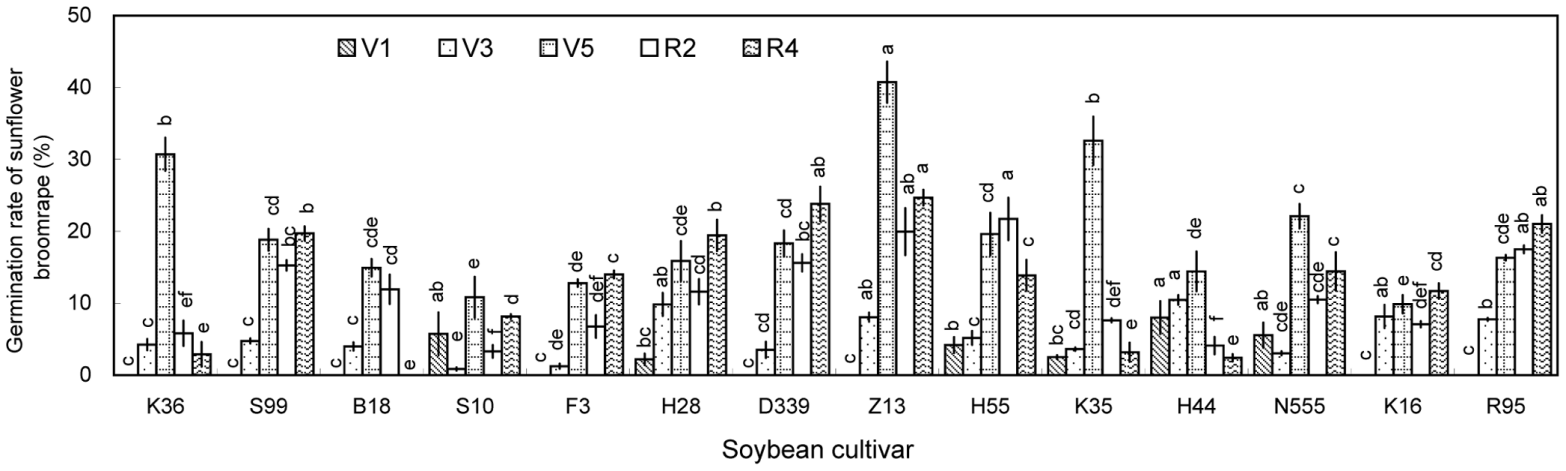
Induction of sunflower broomrape germination by methanolic extracts of soybean rhizosphere soil in pot experiment. The rhizosphere soil was collected from pot-grown plants at the (A) V1, (B) V3, (C) V5, (D) R2, and (E) R4 stages. Error bars represent the standard error of the mean. Different small letters above the error bars indicate significant differences at the 0.05 level (ANOVA and Duncan’s multiple range test). If any letter marked in one treatment is the same as the other one (in same color column), it indicates no significant differences between them. Abbreviations: K36, Kenjiandou 36; S99, Suinong 99; B18, Beidou 18; S10, Suinong 10; F3, Fengdou 3; H28, Heinong 28; D339, Dongdou 339; Z13, Zhonghuang 13; H55, Hefeng 55; K35, Kenjiandou 35; H44, Heinong 44; N555, Nongda 555; K16, Kenfeng 16; R95, Ribenchun 95.

Undiluted aqueous and methanolic extracts of rhizosphere soil generally induced broomrape germination (Fig. 2 and 3), whereas the 10- and 100-fold dilutions induced very low germination (Table S1, S2). Aqueous extracts of rhizosphere soil collected at V3 induced the highest broomrape germination rates, whereas extracts of rhizosphere soil collected at other growth stages induced negligible germination (<10%) (Fig. 2). The differences among the cultivars were significant (*P*<0.05), with aqueous extracts from Suinong 10 (31.4%), Zhonghuang 13 (28.0%), and Heinong 44 (24.4%) inducing the highest germination rates. Methanolic extracts generally induced higher broomrape germination rates than the aqueous extracts (Fig. 3). The highest germination rates were induced by methanolic extracts of rhizosphere soil collected at V5 (Zhonghuang 13, 40.7%; Kenjiandou 35, 32.6%; Kenjiandou 36, 30.7%). Methanolic extracts of rhizosphere soils collected at V1 induced negligible germination (<10%).

#### Soybean tissue extracts

**Figure 4:**
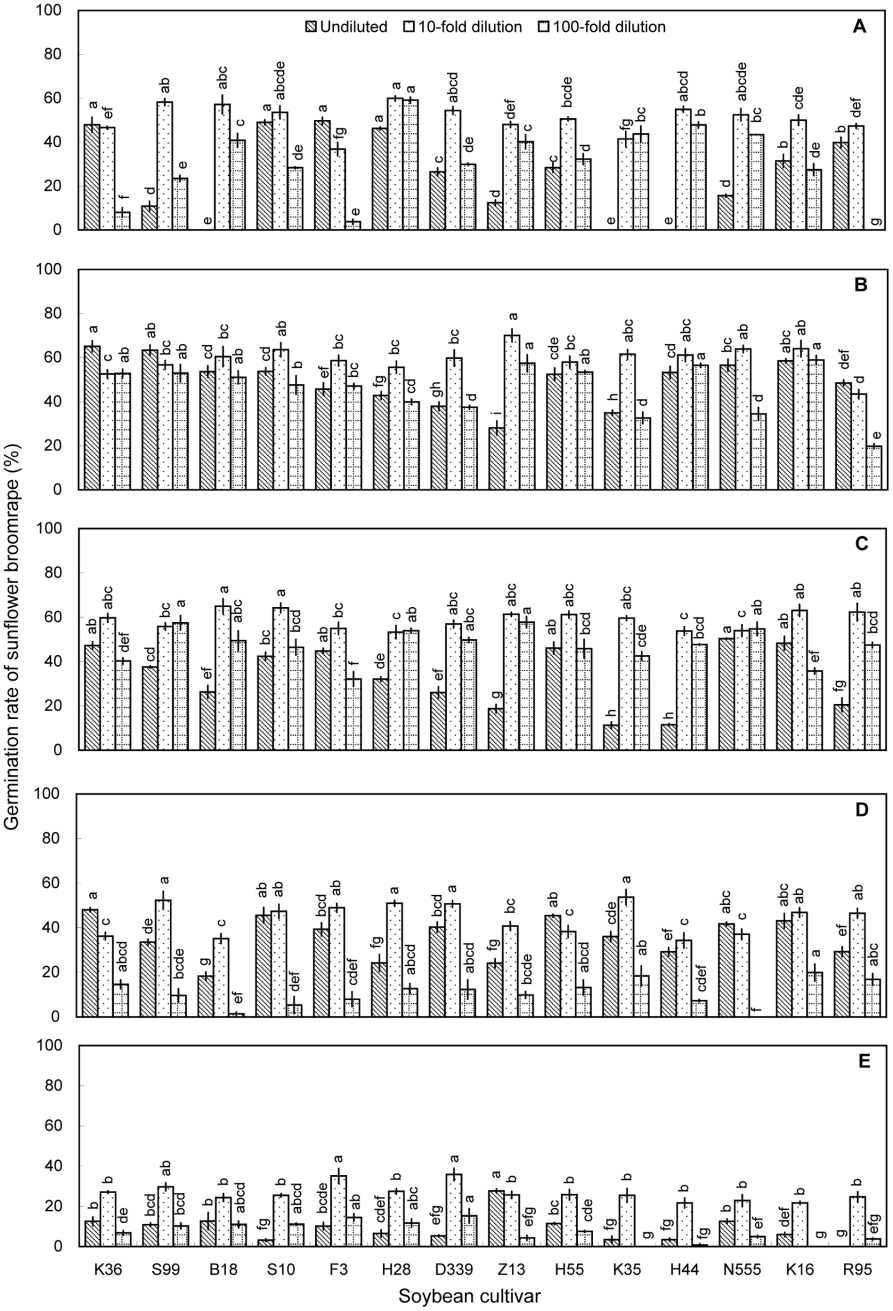
Induction of sunflower broomrape germination by methanolic extracts of soybean roots in pot experiment. Three concentrations of methanolic extracts were assessed, namely, undiluted, 10-fold dilution and 100-fold dilution. The roots were collected from pot-grown plants at the (A) V1, (B) V3, (C) V5, (D) R2, and (E) R4 stages. Error bars represent the standard error of the mean. Different small letters above the error bars indicate significant differences at the 0.05 level (ANOVA and Duncan’s multiple range test). If any letter marked in one treatment is the same as the other one (in same color column), it indicates no significant differences between them. Abbreviations: K36, Kenjiandou 36; S99, Suinong 99; B18, Beidou 18; S10, Suinong 10; F3, Fengdou 3; H28, Heinong 28; D339, Dongdou 339; Z13, Zhonghuang 13; H55, Hefeng 55; K35, Kenjiandou 35; H44, Heinong 44; N555, Nongda 555; K16, Kenfeng 16; R95, Ribenchun 95.

**Figure 5:**
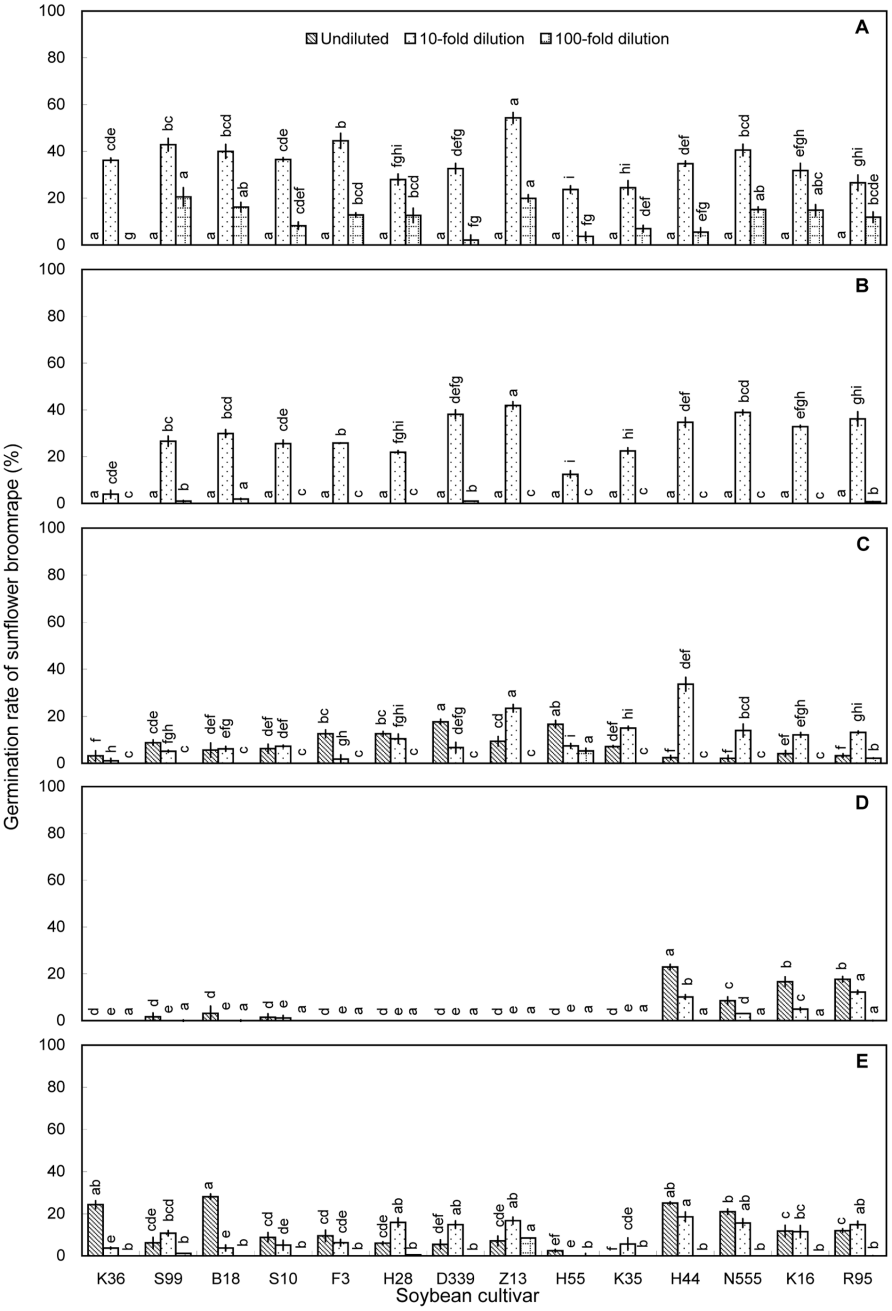
Induction of sunflower broomrape germination by methanolic extracts of soybean stems in pot experiment. Three concentrations of methanolic extracts were assessed, namely, undiluted, 10-fold dilution and 100-fold dilution. The stems were collected from pot-grown plants at the (A) V1, (B) V3, (C) V5, (D) R2, and (E) R4 stages. Error bars represent the standard error of the mean. Different small letters above the error bars indicate significant differences at the 0.05 level (ANOVA and Duncan’s multiple range test). If any letter marked in one treatment is the same as the other one (in same color column), it indicates no significant differences between them. Abbreviations: K36, Kenjiandou 36; S99, Suinong 99; B18, Beidou 18; S10, Suinong 10; F3, Fengdou 3; H28, Heinong 28; D339, Dongdou 339; Z13, Zhonghuang 13; H55, Hefeng 55; K35, Kenjiandou 35; H44, Heinong 44; N555, Nongda 555; K16, Kenfeng 16; R95, Ribenchun 95.

**Figure 6:**
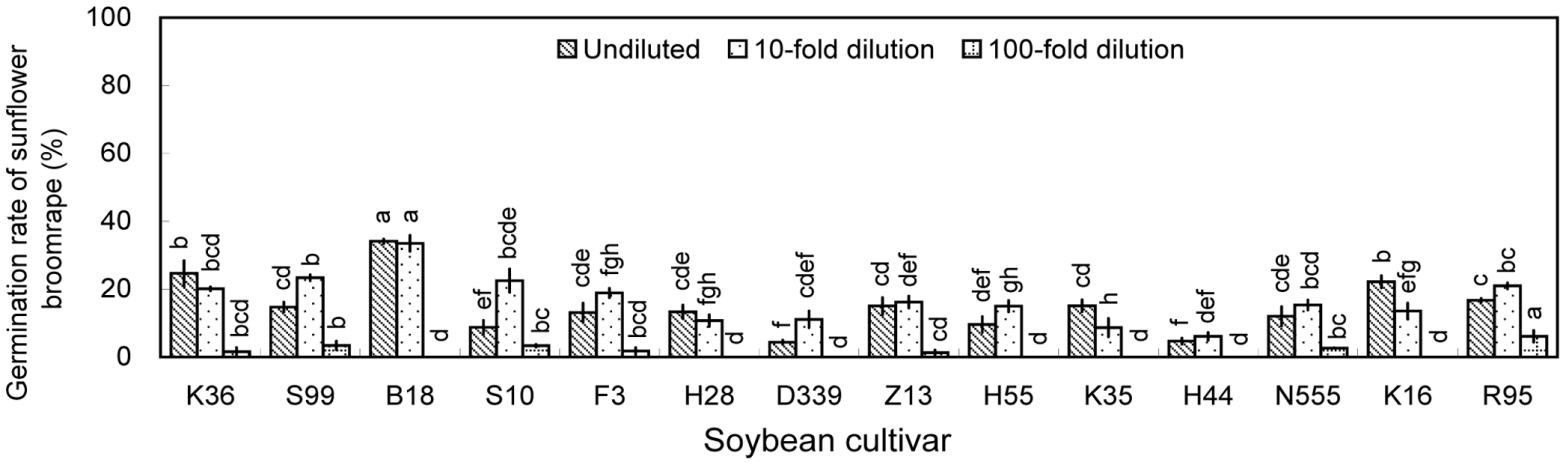
Induction of sunflower broomrape germination by methanolic extracts of soybean leaves collected at the V3 stage in pot experiment. Three concentrations of methanolic extracts were assessed, namely, undiluted, 10-fold dilution and 100-fold dilution. Error bars represent the standard error of the mean. Different small letters above the error bars indicate significant differences at the 0.05 level (ANOVA and Duncan’s multiple range test). If any letter marked in one treatment is the same as the other one (in same color column), it indicates no significant differences between them. Abbreviations: K36, Kenjiandou 36; S99, Suinong 99; B18, Beidou 18; S10, Suinong 10; F3, Fengdou 3; H28, Heinong 28; D339, Dongdou 339; Z13, Zhonghuang 13; H55, Hefeng 55; K35, Kenjiandou 35; H44, Heinong 44; N555, Nongda 555; K16, Kenfeng 16; R95, Ribenchun 95.

Methanolic extracts of soybean roots or shoots (i.e., stems or leaves) generally induced significant broomrape germination (Fig. 4, 5, 6), whereas aqueous extracts induced germination rates of <10% (Table S3, S4, S5). Among methanolic root extracts, extracts of roots collected at V3 induced the highest broomrape germination rates. The ability of root extracts to induce germination generally declined as the plants matured beyond V3. At V3, methanolic root extracts of nine cultivars induced germination rates >60% (Kenjiandou 36, undiluted, 65.2%; Suinong 99, undiluted, 63.3%; Beidou 18, 10-fold dilution, 60.5%; Suinong 10, 10-fold dilution, 63.6%; Zhonghuang 13, 10-fold dilution, 70.1%; Kenjiandou 35, 10-fold dilution, 61.5%; Heinong 44, 10-fold dilution, 61.2%; Nongda 555, 10-fold dilution, 63.9%; and Kenfeng 16, 10-fold dilution, 64.1%). The differences among cultivars were significant (*P*<0.05).

Extracts of soybean stems collected at V1 induced the highest broomrape germination rates. Extracts of stems collected at V5, R2, and R4 induced germination rates <30%. Ten-fold dilutions of stem extracts induced higher germination rates than the undiluted- or 100-fold dilutions. The differences among cultivars were significant (*P*<0.05), with Zhonghuang 13 inducing the highest germination rate (54.3%) (Fig. 5).

Among leaf extracts, extracts of soybean leaves collected at V3 induced the highest germination. Only Beidou 18 induced germination rates >30% (Undiluted, 37.5%; 10-fold dilution, 37.0%) (Fig. 6). Data for leaf extracts collected at V1, V5, R2 and R4 induced negligible germination rates of <10% (Table S6).

Overall, aqueous extracts and methanolic extracts of rhizosphere soils and methanolic extracts of leaves induced relatively low germination rates (Fig. 1, 2, 3, and 6). Methanolic root extracts induced the highest germination (Fig. 4), followed by methanolic stem extracts (Fig. 5).

**Figure 7:**
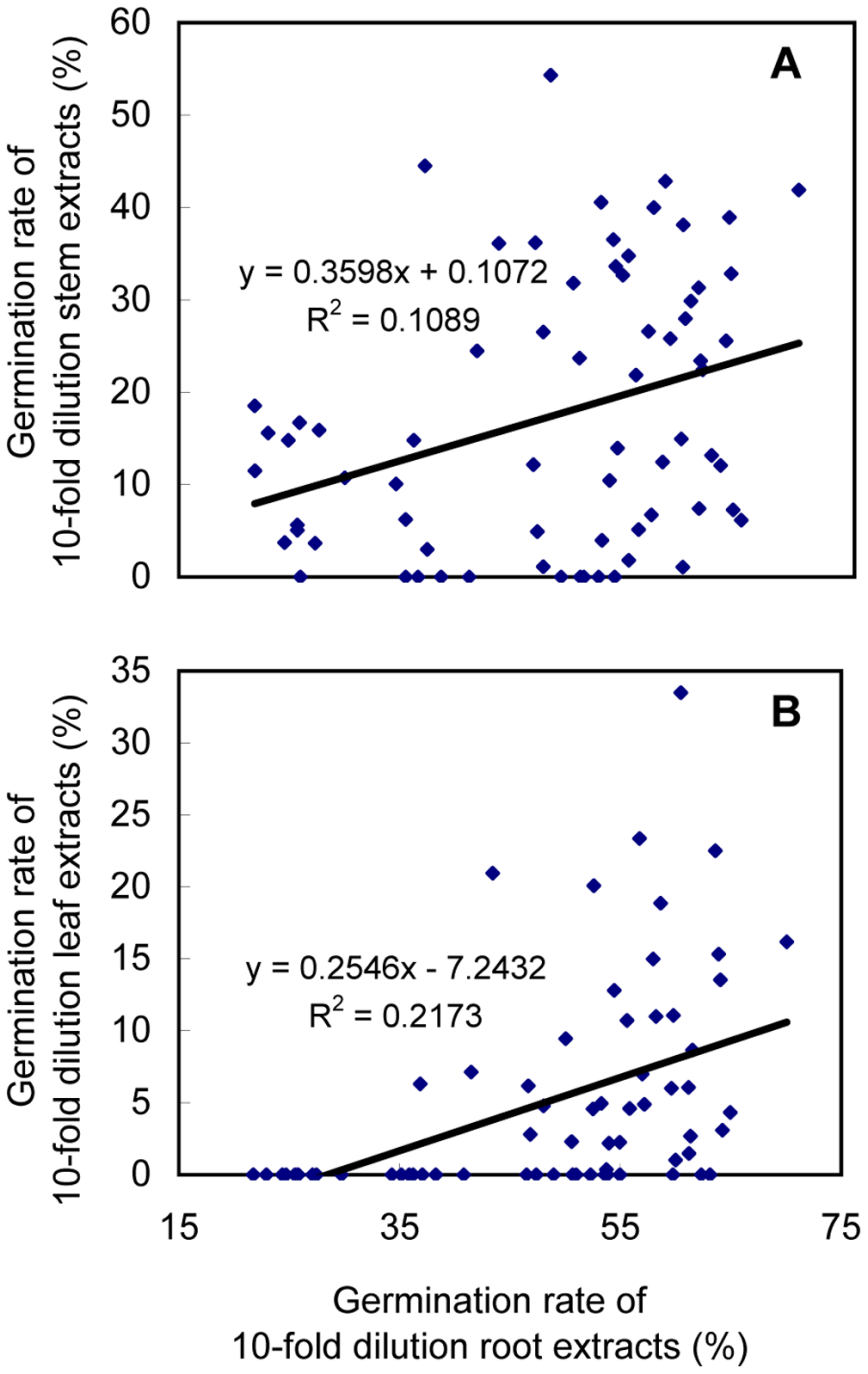
Correlation analysis. Correlation between the germination rates of sunflower broomrape seeds treated with 10-fold dilutions of methanolic root extracts and those treated with (A) 10-fold dilutions of methanolic stem extracts or (B) 10-fold dilutions of methanolic soybean leaf extracts.

Correlation analysis was done using the germination rates induced by different soybean tissues of all 14 cultivars. The germination rates induced by 10-fold dilutions of root extracts were positively correlated with those induced by 10-fold dilutions of stem extracts (Fig. 7A, R ^2^ = 0.1089, *P = *0.005) and 10-fold dilutions of leaf extracts (Fig. 7B, R ^2^ = 0.2173, *P*<0.001).

**Figure 8:**
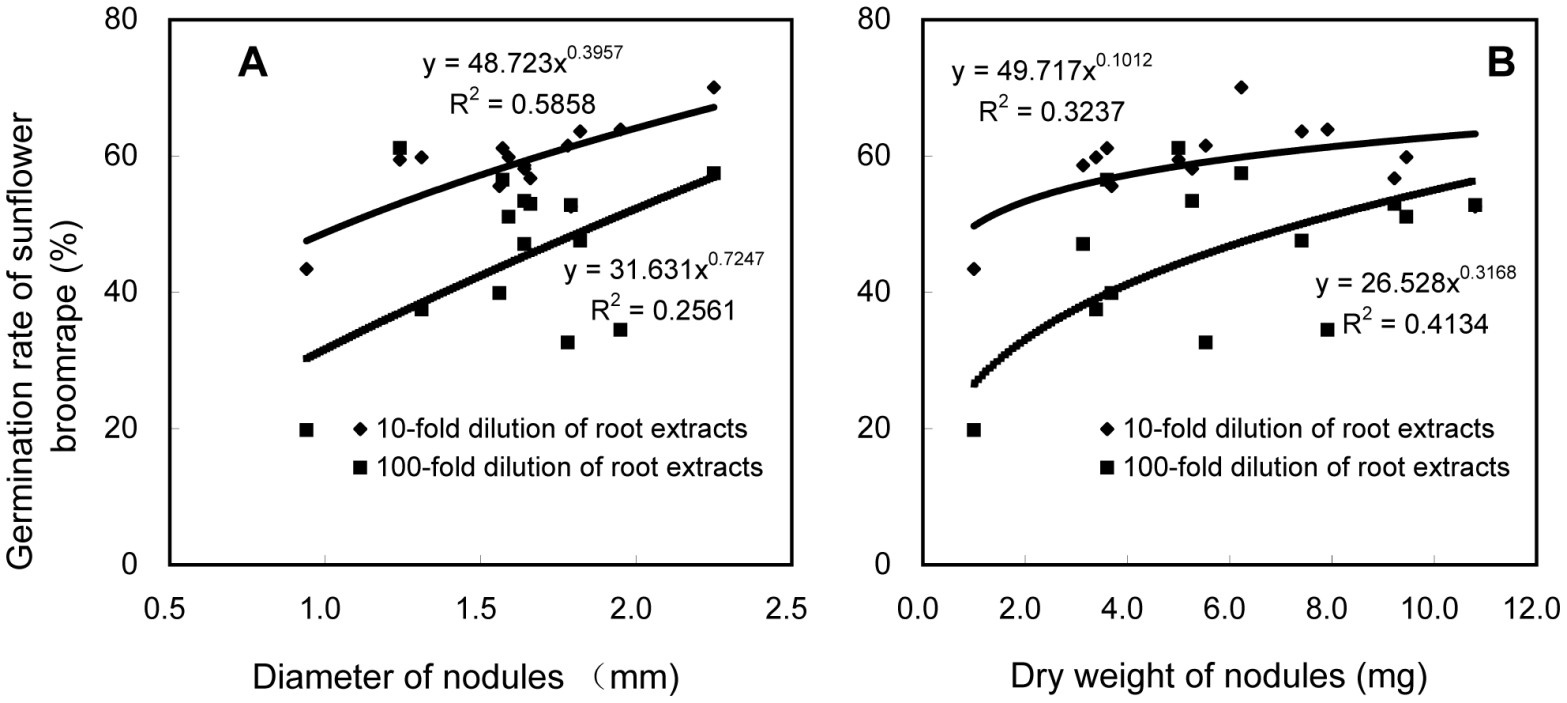
Correlation analysis. Correlation between the germination rates of sunflower broomrape seeds treated with 10- or 100-fold dilutions of methanolic root extracts and (A) soybean nodule diameter and (B) soybean nodule dry weight at V3 stage.

At V3, the 10-fold dilutions of soybean roots extracts stimulated higher germination rates than the 100-fold dilution extracts. The germination rates induced by 10-fold dilutions of root extracts ranged from 43.4 to 70.1%. In comparison, the 100-fold dilutions of root extracts induced germination rates between 19.7 and 61.2%. The diameter of the root nodules ranged from 0.94 to 2.25 cm. The germination rates induced by 10- and 100-fold dilutions of root extracts increased in the form of power as the diameter of the nodules increased (10-fold dilutions, y = 49.717×^0.1012^, R^2^ = 0.3237, *P* = 0.034; 100-fold dilutions, y = 26.528×^0.3168^, R^2^ = 0.4134, *P* = 0.013, Fig. 8A). The dry weight of nodules at V3 ranged from 1.0 to 10.8 g per plant. The germination rates induced by 10- and 100-fold dilutions of root extracts increased in the form of power as the dry weight of root nodules increased (10-fold dilutions, y = 48.723×^0.3957^, R^2^ = 0.5858, *P* = 0.001; 100-fold dilutions, y = 31.631×^0.7247^, R^2^ = 0.2561, *P* = 0.065, Fig. 8B).

### Experiment 2: Field Experiment

**Figure 9:**
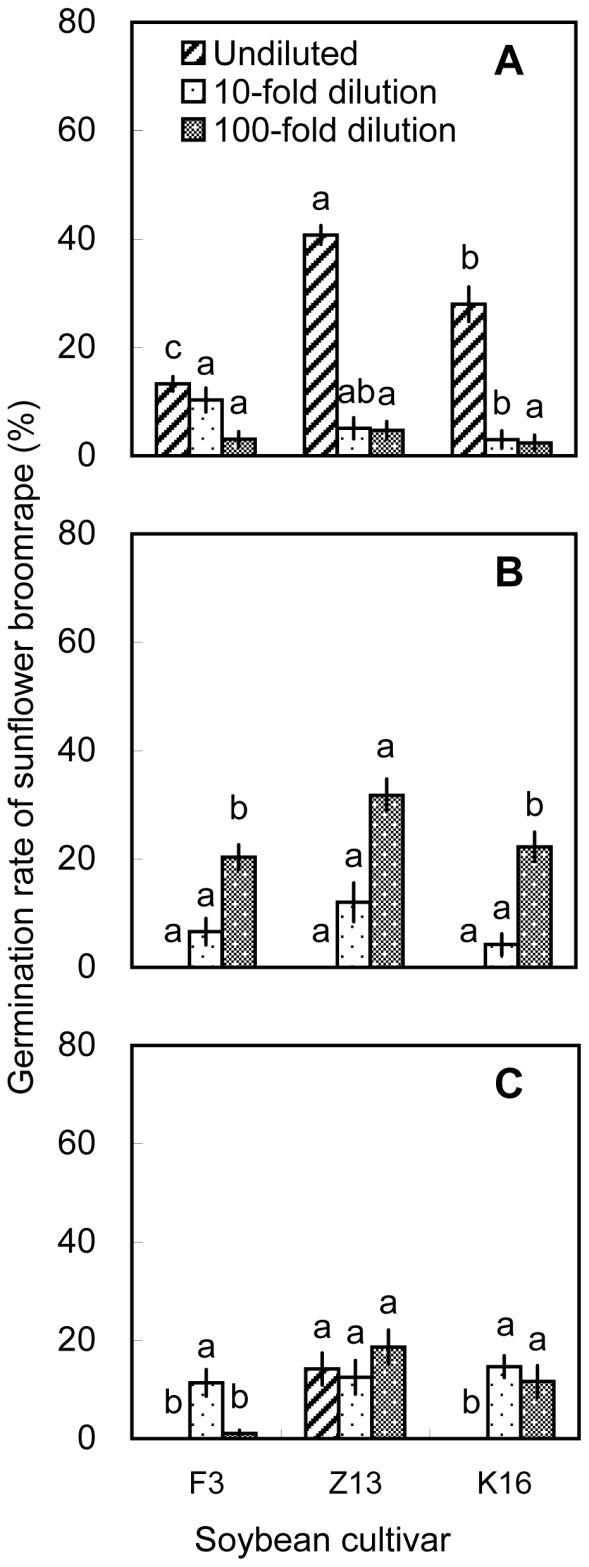
Induction of sunflower broomrape germination by methanolic extracts of soybean rhizosphere soil in field experiment. Three concentrations of methanolic extracts were assessed, namely, undiluted, 10-fold dilution and 100-fold dilution. The rhizosphere soil was collected from field-grown plants at the (A) V3, (B) V5, and (C) R4 stages. Error bars represent the standard error of the mean. Different small letters above the error bars indicate significant differences at the 0.05 level (ANOVA and Duncan’s multiple range test). If any letter marked in one treatment is the same as the other one (in same color column), it indicates no significant differences between them. Abbreviations: F3, Fengdou 3; Z13, Zhonghuang 13; K16, Kenfeng 16.

**Figure 10:**
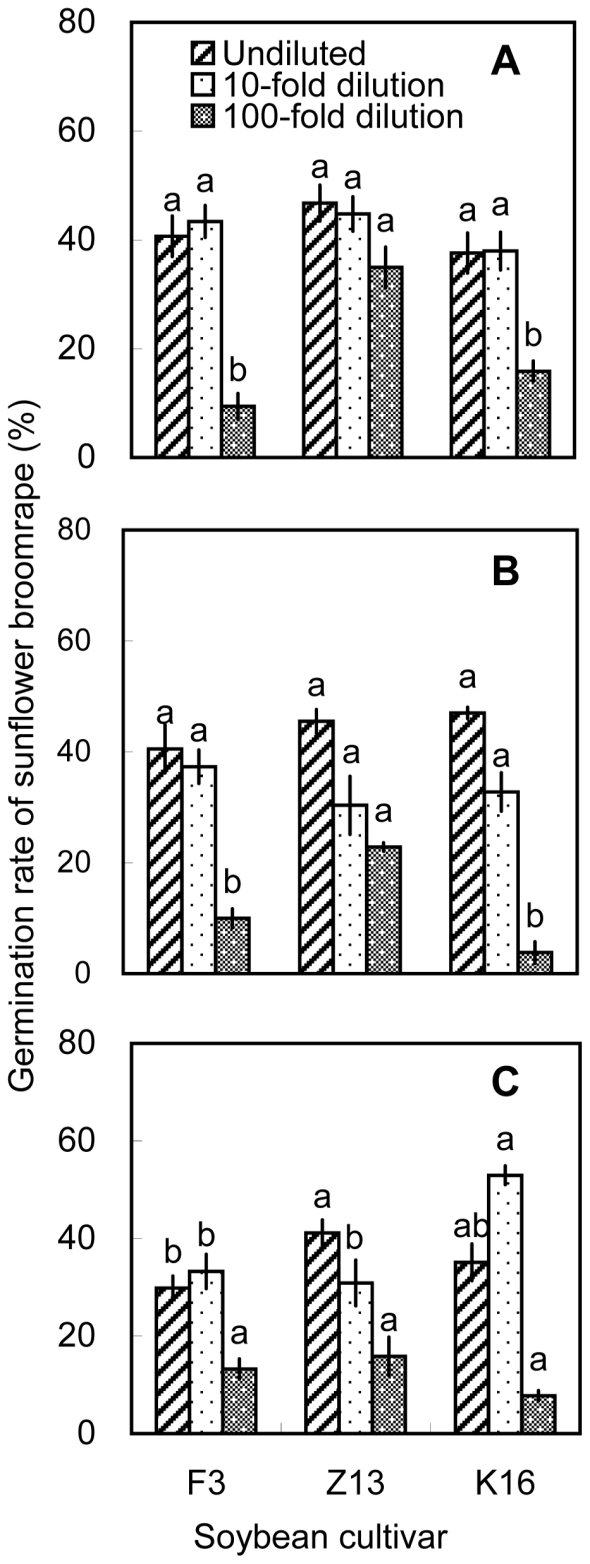
Induction of sunflower broomrape germination by methanolic extracts of soybean roots in field experiment. Three concentrations of methanolic extracts were assessed, namely, undiluted, 10-fold dilution and 100-fold dilution. The roots were collected from field-grown plants at the (A) V3, (B) V5, and (C) R4 stages. Error bars represent the standard error of the mean. Different small letters above the error bars indicate significant differences at the 0.05 level (ANOVA and Duncan’s multiple range test). If any letter marked in one treatment is the same as the other one (in same color column), it indicates no significant differences between them. Abbreviations: F3, Fengdou 3; Z13, Zhonghuang 13; K16, Kenfeng 16.

**Figure 11:**
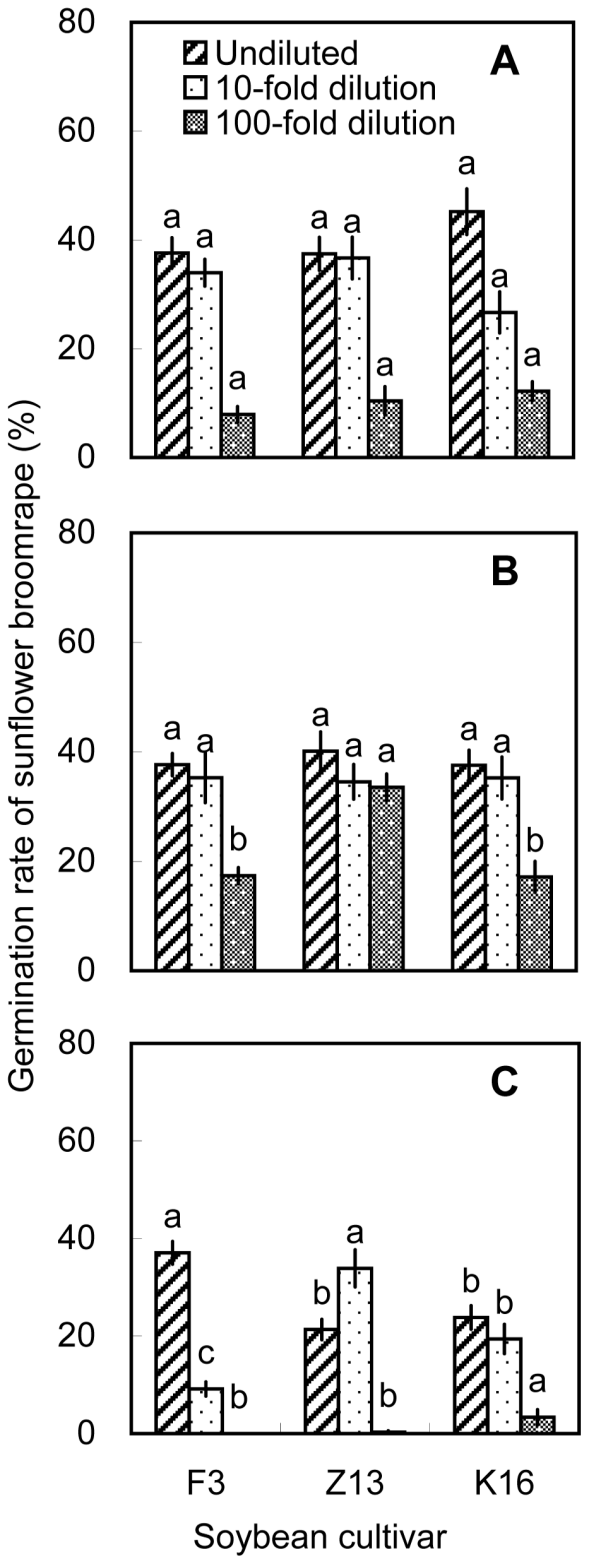
Induction of sunflower broomrape germination by methanolic extracts of soybean stems in field experiment. Three concentrations of methanolic extracts were assessed, namely, undiluted, 10-fold dilution and 100-fold dilution. The stems were collected from field-grown plants at the (A) V3, (B) V5, and (C) R4 stages. Error bars represent the standard error of the mean. Different small letters above the error bars indicate significant differences at the 0.05 level (ANOVA and Duncan’s multiple range test). If any letter marked in one treatment is the same as the other one (in same color column), it indicates no significant differences between them. Abbreviations: F3, Fengdou 3; Z13, Zhonghuang 13; K16, Kenfeng 16.

**Figure 12:**
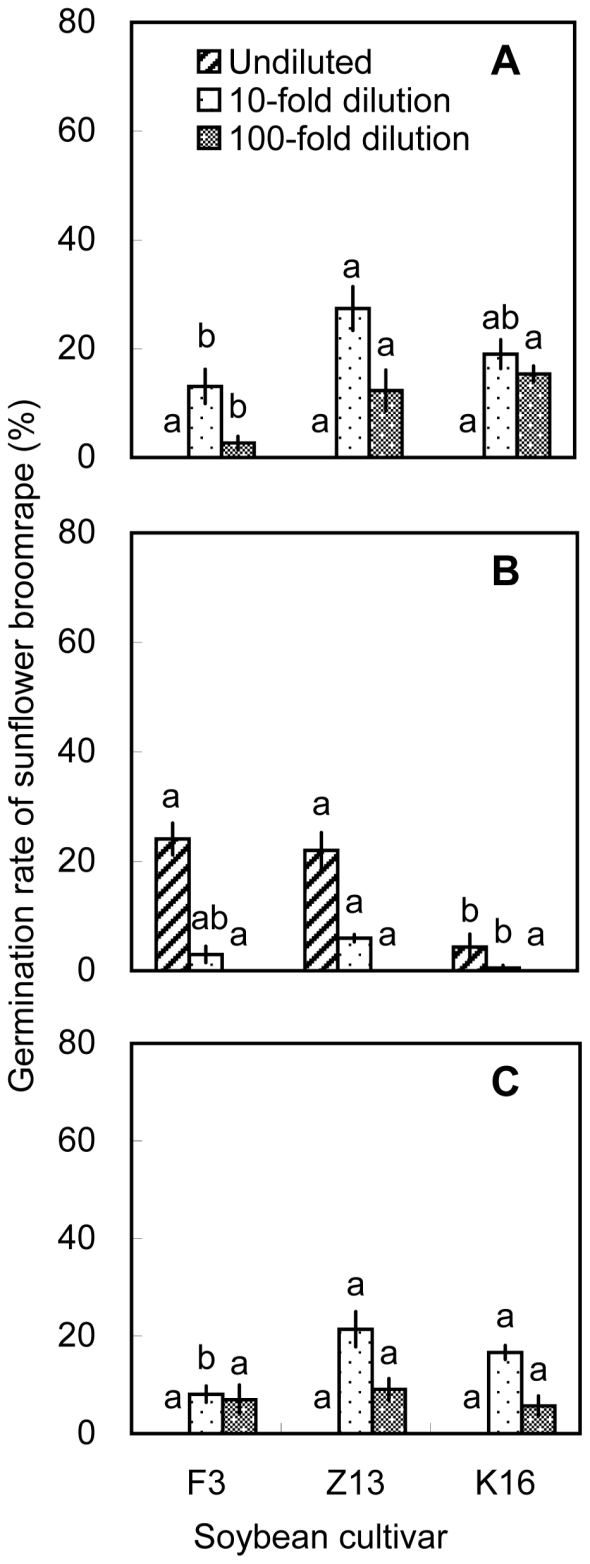
Induction of sunflower broomrape germination by methanolic extracts of soybean leaves in field experiment. Three concentrations of methanolic extracts were assessed, namely, undiluted, 10-fold dilution and 100-fold dilution. The leaves were collected from field-grown plants at the (A) V3, (B) V5, and (C) R4 stages. Error bars represent the standard error of the mean. Different small letters above the error bars indicate significant differences at the 0.05 level (ANOVA and Duncan’s multiple range test). If any letter marked in one treatment is the same as the other one (in same color column), it indicates no significant differences between them. Abbreviations: F3, Fengdou 3; Z13, Zhonghuang 13; K16, Kenfeng 16.

On the basis of the pot experiments, we selected one cultivar with high allelopathic effect (i.e., Zhonghuang 13) and two cultivars with relatively low allelopathic effects (i.e., Fengdou 3 and Kenfeng 16) for a field experiment. The rhizosphere soils and soybean tissues were only extracted with methanol. Undiluted methanolic extracts of rhizosphere soil collected at V3 induced the highest broomrape germination rates. The differences among cultivars were significant (*P*<0.05) with Zhonghuang 13 inducing the highest germination rate (40.8%) at V3. The 100-fold dilutions of rhizosphere soil collected at V5 induced higher broomrape germination rates than the 10-fold and undiluted solutions. Zhonghuang 13 induced the highest germination rate (31.8%, Fig. 9). Methanolic root extracts induced germination rates of >30% at all three growth stages. The 100-fold dilution of methanolic root extracts from Zhonghuang 13 induced the highest germination rate (34.9%) than the other two cultivars, which was consistent with the results of the pot experiment (Fig. 4 and 10). Methanolic stem extracts collected at V3 and V5 induced higher germination rates than methanolic stem extracts collected at R4. Undiluted stem extracts of Kenfeng 16 induced the highest germination rate (45.2%) at V3. However, there was no significant difference among the cultivars (Fig. 11). The germination rates induced by leaf extracts were lower than those induced by stem or root extracts. The differences among the cultivars were significant (*P*<0.05); however the germination rates induced by leaf extracts were <30% (Fig. 12). 10-fold dilution of leaf extracts of Zhonghuang 13 collected at V3 induced higher germination rates than Fengdou 3. Among the undiluted solutions of leaf extracts at V5, those of Fengdou 3 and Zhonghuang 13 induced significantly higher germination rates than those of Kenfeng 16.

## Discussion

Sunflower broomrape is a noxious parasitic weed that has caused huge damage to the farmland ecosystem of China. The possibility of using trap crops to diminish the seed bank of sunflower broomrape has been widely discussed [24]–[26]. One possible trap crop is soybean; however, little is known about the allelopathic effect of soybean on sunflower broomrape. Our results showed that soybean rhizosphere soil can induce sunflower broomrape germination. In the pot experiment, Zhonghuang 13 showed the highest stimulative effects on sunflower broomrape (Fig. 1, 2, and 3). In the field experiment, Zhonghuang 13 showed higher stimulative effects than two other cultivars (Fengdou 3 and Kenfeng 16) (Fig. 9).

Our results indicate that soybeans produce the germination stimulant required by sunflower broomrape. This agrees with the previous report that soybean root exudates contained the orobanchyl acetate, which is one of the strigolactones [15]. The type and concentration of allelopathic substances may differ among plant organs [27]. In our study, root extracts induced higher broomrape germination rates than stem or leaf extracts. This is in consistent with the opinion that strigolactones are mainly synthesized in roots and transported to shoots [28]. Similar observations have been made for red clover (*Trifolium pratense* L.), cotton (*Gossypium hirsutum* L.), and tobacco (*Nicotiana tabacum* L.) [29]–[32]. Correlation analysis confirmed significant correlation among germination rates induced by soybean roots, stem, and leaf extracts (Fig. 7).

Methanolic extracts generally induced broomrape germination whereas aqueous extracts generally did not. This indicates that the chemical composition of the aqueous and methanolic extracts was not the same. Undiluted stem and leaf extracts generally induced lower germination than the 10-fold or 100-fold dilutions (Fig. 5, 6). One explanation is that the extracts may contain compounds that inhibit broomrape germination [33]. If this is true, then perhaps the concentration of these compounds was too low to inhibit germination in the 10- and 100-fold dilutions. Additional experiments need to be done to confirm this hypothesis.

An et al. [34] observed that allelochemical concentrations in some plants change across time. Specifically, those authors observed that root extracts of the red clover (*Trifolium pratense* L.) can induce clover broomrape germination when the samples were collected from the first to fifth trifoliate stage. And root exudates of red clover induced maximum germination at the third trifoliate stage. For wheat (*Triticum aestivum* L.), maximum germination was induced by root exudates of wheat seedlings. Root exudates of wheat plants beyond tillering induced minimum germination [35]. We observed that the allelopathic effects of soybeans towards broomrape generally peaked at V3 and then declined gradually (Fig. 4). Therefore, the soybean at V3 stage produced the greatest sunflower broomrape germination stimulant.

The soybean cultivars differed in their ability to induce sunflower broomrape germination (Fig. 4). This is similar to previous reports that the production of germination stimulants varied among wheat cultivars and among cotton genotypes [11], [36]. In our pot experiment, Kenfeng 16, Zhonghuang 13, and Beidou 18 induced significantly higher broomrape germination than the other cultivars. In the field experiment, Zhonghuang 13 induced higher broomrape germination than Fengdou 3 and Kenfeng 16.

Positive correlations existed between the broomrape germination and soybean nodule diameter and dry weight. This supports the hypothesis that endogenous strigolactones may promote nodulation in soybean as it does in pea (*Pisum sativum* L.) [37]. We suggest that soybean cultivars with many root nodules may have the best potential for managing broomrape.

This study demonstrated that commonly grown soybean cultivars have the potential to reduce the seed bank of sunflower broomrape. Cultivars may vary in allelopathic effects toward sunflower broomrape. Methanolic extracts of soybean roots induced higher broomrape germination rates than extracts of stems or leaves. The allelopathic effect of soybean was greatest at the V3 stage. Further research should be done in sunflower broomrape infested fields to confirm the efficacy of using soybean as a trap crop under field conditions. Trap crops probably cannot completely eliminate the sunflower broomrape seed bank in a single life cycle; therefore, integrated management of sunflower broomrape is needed.

## Supporting Information

Table S1 **Sunflower broomrape seeds germination induced by distilled water extracts (10- and 100-fold dilution) of soybean rhizosphere soils at different stages in pot experiment (%).**
(DOC)

Table S2 **Sunflower broomrape seeds germination induced by methanolic extracts (10- and 100-fold dilution) of soybean rhizosphere soils at different stages in pot experiment (%).**
(DOC)

Table S3 **Sunflower broomrape seeds germination induced by distilled water extracts of soybean roots at different stages in pot experiment (%).**
(DOC)

Table S4 **Sunflower broomrape seeds germination induced by distilled water extracts soybean stems at different stages in pot experiment (%).**
(DOC)

Table S5 **Sunflower broomrape seeds germination induced by distilled water extracts of soybean leaves at different stages in pot experiment (%).**
(DOC)

Table S6 **Sunflower broomrape seeds germination induced by methanolic extracts of soybean leaves at V1, V5, R2 and R4 stages in pot experiment (%).**
(DOC)
