# Supplementary material for: Some Soybean Cultivars Have Ability to Induce Germination of Sunflower Broomrape
Source: PLoS One. 2013 Mar 27;8(3):e59715. doi: 10.1371/journal.pone.0059715 (PMC3609756; doi:10.1371/journal.pone.0059715)
Supplement: Table S2 — Sunflower broomrape seeds germination induced by methanolic extracts (10- and 100-fold dilution) of soybean rhizosphere soils at different stages in pot experiment (%). (DOC) [file pone.0059715.s002.doc]

**Table S2 Sunflower broomrape seeds germination induced by methanolic extracts (10- and 100-fold dilution) of soybean rhizosphere soils at different stages in pot experiment (%)**

| **Varieties** | **Growth Stages** | | | | | | | | | |
| --- | --- | --- | --- | --- | --- | --- | --- | --- | --- | --- |
|  | **V1** | | **V3** | | **V5** | | **R2** | | **R4** | |
|  | **1%** | **10%** | **1%** | **10%** | **1%** | **10%** | **1%** | **10%** | **1%** | **10%** |
| Kenjiandou 36 | 0.0 a | 0.0 a | 0.0 a | 0.0 a | 0.0 a | 0.0 a | 0.0 a | 0.0 a | 0.0 a | 0.0 a |
| Suinong 99 | 0.0 a | 0.0 a | 0.0 a | 0.0 a | 0.0 a | 0.0 a | 0.0 a | 0.0 a | 0.0 a | 0.0 a |
| Beidou 18 | 0.0 a | 0.0 a | 0.0 a | 0.0 a | 0.0 a | 4.1 a | 0.0 a | 0.0 a | 0.0 a | 0.0 a |
| Suinong 10 | 0.0 a | 2.2 a | 0.0 a | 0.0 a | 0.0 a | 0.0 a | 0.0 a | 0.0 a | 0.0 a | 0.0 a |
| Fengdou 3 | 0.0 a | 0.0 a | 2.8 a | 0.0 a | 0.0 a | 0.0 a | 0.0 a | 0.0 a | 0.0 a | 0.0 a |
| Heinong 28 | 0.0 a | 4.8 a | 0.0 a | 0.0 a | 0.0 a | 3.3 a | 0.0 a | 1.3 a | 0.0 a | 3.2 a |
| Dongdou 339 | 0.0 a | 0.0 a | 0.0 a | 0.0 a | 0.0 a | 0.0 a | 0.0 a | 0.0 a | 0.0 a | 0.0 a |
| Zhonghuang 13 | 0.0 a | 1.9 a | 0.0 a | 1.7 a | 0.0 a | 0.0 a | 0.0 a | 0.0 a | 0.0 a | 3.5 a |
| Hefeng 55 | 0.0 a | 0.0 a | 2.2 a | 0.0 a | 0.0 a | 0.0 a | 0.0 a | 0.0 a | 0.0 a | 0.0 a |
| Kenjiandou 35 | 0.0 a | 1.5 a | 0.0 a | 0.0 a | 0.0 a | 6.2 a | 0.0 a | 0.0 a | 0.0 a | 0.0 a |
| Heinong 44 | 0.0 a | 0.0 a | 3.4 a | 0.0 a | 0.0 a | 3.3 a | 0.0 a | 0.0 a | 0.0 a | 0.0 a |
| Nongda 555 | 0.0 a | 0.0 a | 0.0 a | 0.0 a | 0.0 a | 0.0 a | 0.0 a | 0.0 a | 0.0 a | 0.0 a |
| Kenfeng 16 | 0.0 a | 0.0 a | 0.0 a | 0.0 a | 0.0 a | 0.0 a | 0.0 a | 0.0 a | 0.0 a | 0.0 a |
| Ribenchun 95 | 0.0 a | 0.0 a | 0.0 a | 0.0 a | 0.0 a | 0.0 a | 0.0 a | 0.0 a | 0.0 a | 0.0 a |

1%: 100-fold dilution (0.05 g/mL); 10%: 10-fold dilution (0.005 g/mL).
