# Supplementary material for: Some Soybean Cultivars Have Ability to Induce Germination of Sunflower Broomrape
Source: PLoS One. 2013 Mar 27;8(3):e59715. doi: 10.1371/journal.pone.0059715 (PMC3609756; doi:10.1371/journal.pone.0059715)
Supplement: Table S3 — Sunflower broomrape seeds germination induced by distilled water extracts of soybean roots at different stages in pot experiment (%). (DOC) [file pone.0059715.s003.doc]

**Table S3 Sunflower broomrape seeds germination induced by distilled water extracts of soybean roots at different stages in pot experiment (%)**

| **Varieties** | **Growth Stages** | | | | | | | | | | | | | | |
| --- | --- | --- | --- | --- | --- | --- | --- | --- | --- | --- | --- | --- | --- | --- | --- |
|  | **V1** | | | **V3** | | | **V5** | | | **R2** | | | **R4** | | |
|  | **1%** | **10%** | **100%** | **1%** | **10%** | **100%** | **1%** | **10%** | **100%** | **1%** | **10%** | **100%** | **1%** | **10%** | **100%** |
| Kenjiandou 36 | 5.5 ab | 0.0 a | 0.0 a | 4.2 ab | 0.0 b | 0.0 a | 1.2 a | 4.2 ab | 0.0 a | 0.0 a | 0.0 a | 0.0 a | 0.0 a | 0.0 a | 0.0 a |
| Suinong 99 | 4.6 ab | 3.0 a | 0.0 a | 7.0 a | 0.7 b | 0.0 a | 0.0 a | 7.0 a | 0.0 a | 0.0 a | 0.0 a | 0.0 a | 0.0 a | 0.0 a | 0.0 a |
| Beidou 18 | 4.9 ab | 0.0 a | 0.0 a | 6.0 a | 0.0 b | 0.0 a | 0.0 a | 6.0 a | 0.0 a | 0.0 a | 0.0 a | 0.0 a | 0.0 a | 0.0 a | 0.0 a |
| Suinong 10 | 5.0 ab | 1.0 a | 0.0 a | 5.7 a | 2.1 b | 0.0 a | 0.0 a | 5.7 a | 0.0 a | 0.0 a | 0.0 a | 0.0 a | 0.0 a | 0.0 a | 0.0 a |
| Fengdou 3 | 4.9 ab | 1.2 a | 0.0 a | 7.3 a | 7.1 a | 0.0 a | 0.0 a | 7.3 a | 0.0 a | 0.0 a | 0.0 a | 0.0 a | 0.0 a | 0.0 a | 0.0 a |
| Heinong 28 | 4.2 ab | 1.2 a | 0.0 a | 5.3 a | 3.1 b | 0.0 a | 0.0 a | 5.3 a | 0.0 a | 0.0 a | 0.0 a | 0.0 a | 0.0 a | 0.0 a | 0.0 a |
| Dongdou 339 | 7.5 a | 5.0 a | 0.0 a | 7.5 a | 0.0 b | 0.0 a | 0.0 a | 7.5 a | 0.0 a | 0.0 a | 0.0 a | 0.0 a | 0.0 a | 0.0 a | 0.0 a |
| Zhonghuang 13 | 5.3 ab | 2.5 a | 0.0 a | 4.5 ab | 0.0 b | 0.0 a | 0.0 a | 4.5 ab | 0.0 a | 0.0 a | 0.0 a | 0.0 a | 0.0 a | 0.0 a | 0.0 a |
| Hefeng 55 | 4.4 ab | 3.3 a | 0.0 a | 4.5 ab | 0.0 b | 0.0 a | 0.0 a | 4.5 ab | 0.0 a | 0.0 a | 3.3 a | 0.0 a | 0.0 a | 0.0 a | 0.0 a |
| Kenjiandou 35 | 3.5 ab | 2.5 a | 0.0 a | 0.0 b | 0.0 b | 0.0 a | 0.0 a | 0.0 b | 0.0 a | 0.0 a | 0.0 a | 0.0 a | 0.0 a | 0.0 a | 0.0 a |
| Heinong 44 | 0.4 b | 1.9 a | 0.0 a | 6.0 a | 0.0 b | 0.0 a | 0.0 a | 6.0 a | 0.0 a | 0.0 a | 4.2 a | 0.0 a | 0.0 a | 0.0 a | 0.0 a |
| Nongda 555 | 2.7 ab | 2.6 a | 0.0 a | 3.5 ab | 0.0 b | 0.0 a | 3.1 a | 3.5 ab | 0.0 a | 0.0 a | 0.0 a | 0.0 a | 0.0 a | 0.0 a | 0.0 a |
| Kenfeng 16 | 2.9 ab | 0.0 a | 0.0 a | 5.4 a | 0.0 b | 0.0 a | 2.2 a | 5.4 a | 0.0 a | 0.0 a | 0.0 a | 0.0 a | 0.0 a | 0.0 a | 0.0 a |
| Ribenchun 95 | 4.6 ab | 2.0 a | 0.0 a | 0.0 b | 2.8 b | 0.0 a | 0.0 a | 0.0 b | 0.0 a | 0.0 a | 0.0 a | 0.0 a | 0.0 a | 0.0 a | 0.0 a |

1%: 100-fold dilution (0.01 g/mL); 10%: 10-fold dilution (0.1 g/mL); 100%: undiluted solution (1 g/mL).
