# Supplementary material for: Some Soybean Cultivars Have Ability to Induce Germination of Sunflower Broomrape
Source: PLoS One. 2013 Mar 27;8(3):e59715. doi: 10.1371/journal.pone.0059715 (PMC3609756; doi:10.1371/journal.pone.0059715)
Supplement: Table S6 — Sunflower broomrape seeds germination induced by methanolic extracts of soybean leaves at V1, V5, R2 and R4 stages in pot experiment (%). (DOC) [file pone.0059715.s006.doc]

**Table S6 Sunflower broomrape seeds germination induced by methanolic extracts of soybean leaves at V1, V5, R2 and R4 stages in pot experiment (%)**

| **Varieties** | **Growth Stages** | | | | | | | | | | | |
| --- | --- | --- | --- | --- | --- | --- | --- | --- | --- | --- | --- | --- |
|  | **V1** | | | **V5** | | | **R2** | | | **R4** | | |
|  | **1%** | **10%** | **100%** | **1%** | **10%** | **100%** | **1%** | **10%** | **100%** | **1%** | **10%** | **100%** |
| Kenjiandou 36 | 0.0 a | 6.2 abc | 0.0 c | 0.0 a | 0.0 f | 0.0 a | 0.0 a | 0.0 b | 0.0 a | 0.0 a | 0.0 a | 0.0 a |
| Suinong 99 | 0.0 a | 6.0 abc | 0.6 bc | 0.0 a | 4.6 bc | 0.0 a | 0.0 a | 0.0 b | 0.0 a | 0.0 a | 0.0 a | 0.0 a |
| Beidou 18 | 0.0 a | 4.8 bcd | 1.4 abc | 0.0 a | 4.3 bcd | 0.0 a | 0.0 a | 0.0 b | 0.0 a | 0.0 a | 0.0 a | 0.0 a |
| Suinong 10 | 0.0 a | 0.0 e | 1.2 bc | 0.0 a | 3.1 cde | 0.0 a | 0.0 a | 0.0 b | 0.0 a | 0.0 a | 0.0 a | 0.0 a |
| Fengdou 3 | 0.0 a | 6.3 abc | 0.8 bc | 0.0 a | 0.0 f | 0.0 a | 0.0 a | 0.0 b | 0.0 a | 0.0 a | 0.0 a | 0.0 a |
| Heinong 28 | 0.0 a | 1.0 de | 5.1 a | 0.0 a | 5.0 abc | 0.0 a | 0.0 a | 0.0 b | 0.0 a | 0.0 a | 0.0 a | 0.0 a |
| Dongdou 339 | 0.0 a | 7.8 ab | 4.1 ab | 0.0 a | 7.0 a | 0.0 a | 0.0 a | 0.0 b | 0.0 a | 0.0 a | 0.0 a | 0.0 a |
| Zhonghuang 13 | 0.0 a | 4.8 bcd | 0.0 c | 0.0 a | 2.7 cde | 0.0 a | 0.0 a | 0.0 b | 0.0 a | 0.0 a | 0.0 a | 0.0 a |
| Hefeng 55 | 0.0 a | 2.3 cde | 0.0 c | 0.0 a | 1.5 ef | 0.0 a | 0.0 a | 0.0 b | 0.0 a | 0.0 a | 0.0 a | 0.0 a |
| Kenjiandou 35 | 0.0 a | 7.1 ab | 0.0 c | 0.0 a | 6.0 ab | 0.0 a | 0.0 a | 0.4 b | 0.0 a | 0.0 a | 0.0 a | 0.0 a |
| Heinong 44 | 0.0 a | 2.3 cde | 0.0 c | 0.0 a | 0.0 f | 0.0 a | 0.0 a | 0.0 b | 0.0 a | 0.0 a | 0.0 a | 0.0 a |
| Nongda 555 | 0.0 a | 4.6 bcd | 1.0 bc | 0.0 a | 2.2 def | 0.0 a | 0.0 a | 0.0 b | 0.0 a | 0.0 a | 0.0 a | 0.0 a |
| Kenfeng 16 | 0.0 a | 9.5 a | 0.0 c | 0.0 a | 0.0 f | 0.0 a | 0.0 a | 2.8 a | 0.0 a | 0.0 a | 0.0 a | 0.0 a |
| Ribenchun 95 | 0.0 a | 0.0 e | 0.0 c | 0.0 a | 0.0 f | 0.0 a | 0.0 a | 0.0 b | 0.0 a | 0.0 a | 0.0 a | 0.0 a |

1%: 100-fold dilution (0.01 g/mL); 10%: 10-fold dilution (0.1 g/mL); 100%: undiluted solution (1 g/mL).
